# Validation of the Dimensions of Anger Reactions Scale (the DAR-5) in non-clinical South Korean adults

**DOI:** 10.1186/s40359-023-01084-8

**Published:** 2023-03-16

**Authors:** Hae Jin Kim, Dong Hun Lee, Jeong Han Kim, Su-Eun Kang

**Affiliations:** 1 Traumatic Stress Center, Department of Education, Sungkyunkwan University, Seoul, Republic of Korea; 2 School of Rehabilitation Services and Counseling, University of Texas-Rio Grande Valley, Edinburg, TX USA

**Keywords:** Adults, DAR-5, Posttraumatic anger, South Korea, Validation

## Abstract

**Background:**

Posttraumatic anger is a commonly reported emotion among people who have experienced traumatic events. The current study aimed to demonstrate the reliability and validity of the South Korean version of the DAR-5 (DAR-5-K). The DAR-5 is a single scale with 5 items which measures posttraumatic anger. The DAR-5 is composed of five items that measure anger frequency, intensity, duration, aggression, and its interference with social relations.

**Methods:**

Data were collected from 814 South Korean adults who had experienced traumatic events and participated in the study and analyzed via the combination of exploratory factor analysis (*n* = 405) and confirmatory factor analysis (n = 409).

**Results:**

Results supported the one-factor structure, as reported in previous validation studies. The scale demonstrated robust internal reliability and concurrent validity with measures of posttraumatic stress disorder (PTSD) symptoms, depression, anxiety, and self-esteem. The DAR-5 cut-off score of 12 that was established in the original validation study successfully differentiated high from low scorers with regard to PTSD symptoms, depression, anxiety, and self-esteem.

**Conclusion:**

The results confirm that the DAR-5-K is a brief and psychometrically robust measure of anger that can be used to examine South Korean adults who have experienced traumatic events.

Anger is a normal emotion that has functional value, such as facilitating physical and psychological resources or promoting perseverance when faced with difficult situations [1, 2]. However, anger can become maladaptive, and problematic anger can be evaluated by assessing its frequency, intensity, duration, aggression, and interference with social functioning [3].

The presence of anger has been identified in a variety of populations who have experienced traumatic events, including combat veterans [4, 5], Cambodian refugees [6], 9/11 disaster relief workers [7], and those who have faced gross human rights violations [8]. Additionally, populations exposed to individual-level traumas would include victims of domestic violence [9], childhood maltreatment [10], and sexual or nonsexual assault [11]. Anger also increases the risk of developing posttraumatic stress disorder (PTSD) symptoms [12, 13]. Thus, reliable and valid measures of anger can be highly useful for evaluating the presence of maladaptive anger in people who have experienced traumatic events.

Several traumatic events have occurred in South Korea recently. In 2003, 192 people lost their lives and 148 were injured due to a fire set by an arsonist in the Daegu Metropolitan Subway. A six-year follow-up study showed that approximately 46.6% of the individuals who were injured in this accident received a PTSD diagnosis and reported adaptive problems in interpersonal relationships and increased vulnerability to stress [14]. A humidifier disinfectant that was potentially lethal when inhaled had been sold widely in South Korea from 1995 to 2011. It took several years for the public to realize the potential danger that the disinfectant’s constituent toxic chemicals posed and for the manufacturer to offer an official apology. Only in 2011 were all the aforementioned disinfectants forcibly recalled by the government. The humidifier disinfectant is reported to have caused serious lung diseases and other medical illnesses in more than 7,000 victims, 1,650 of whom resultantly died [15]. A study that examined 100 families that were harmed by the toxic disinfectant revealed that about 66.6% of them reported a chronic level of posttraumatic embitterment symptoms. In all, 57.5% of the victims reported the emergence of depression/demoralization, 54.3% reported anxiety/nervousness, and 54.3% reported experiencing anger [16].

In 2014, 304 people, 250 of whom were high-school students, lost their lives in one of South Korea’s most distressing maritime tragedies—the Sewol Ferry disaster. Several studies were conducted after the tragedy to identify changes in psychological symptoms among the bereaved families and friends as well as the general public in South Korea, who were indirectly exposed to the event through the media. In addition to the parents of the adolescent victims of the tragedy, the adolescent friends of the deceased students and even middle- and high-school students who did not personally know any of the victims reported emotional changes, which included the emergence of depressive moods and anger [17–19]. In 2017, a 5.4 magnitude earthquake hit Pohang—a mid-sized city located in the southeastern part of South Korea—and resulted in injuries to 135 people. A one-year follow-up study indicated that 13.85% of the 195 survivors who resided in the affected area reported symptoms of posttraumatic stress [20]. More recent traumatic events include a bushfire in a northeastern region of South Korea in 2019, which resulted in two deaths and affected more than 4,000 residents. Numerous studies have been conducted in response to these traumatic events that occurred in South Korea in order to investigate the victims’ posttraumatic symptoms, such as posttraumatic anger.

## Measurement issues in studies on posttraumatic anger in South Korea

Given that a significant number of traumatic events have occurred in South Korea, the need for localized posttraumatic anger studies has been dramatically increasing. In this respect, the lack of cross-culturally validated posttraumatic anger measures is an urgent issue in South Korea. This consequently underscores the importance of a South Korean-validated assessment of posttraumatic anger as an ensuing consequence of various traumatic events, which range from human-induced traumas to natural disasters.

Currently, three instruments are widely used as self-report measures of anger: the State Trait Anger Expression Inventory-2 (STAXI-2) [21], Novaco Anger Scale-Provocation Inventory (NAS-PI) [22], and Dimensions of Anger Reactions Scale (DAR) [23]. The STAXI-2 is a 57-item inventory that measures the experience of anger across several dimensions: state anger (15 items), trait anger (10 items), and four facets of anger expression and control (32 items; expression-out, expression-in, control-out, and control-in) [21]. The NAS-PI consists of two parts: the NAS and PI. The NAS is composed of 60 items that measure cognitive, arousal, and behavioral domains of anger and anger regulation, and the PI contains 25 items that assess anger intensity by describing potentially anger-provoking situations [22].

Although both the STAXI-2 and NAS-PI have been shown to be psychometrically sound measures of anger [21, 22], they are too lengthy to be incorporated into a battery of self-report measures. In contrast, the DAR is a seven-item scale. It consists of four items that measure anger response, which include anger frequency, duration, intensity, and expression, and three items that assess impairment in work performance, social relationships, and health [23]. Forbes et al. [1] validated the DAR with combat veterans. Subsequently, Forbes et al. [3] developed the DAR-5—a shortened version of DAR that uses only five items from it (excluding two items related to impairment in work performance and health). The DAR-5 should offer greater accessibility in disaster situations in comparison to the other aforementioned measures of anger as it is brief and largely reduces the cognitive burden on respondents. Therefore, the current study utilized the DAR-5.

As a result of previous studies, the DAR-5 has been mainly used to measure anger levels after experiencing various types of traumatic events worldwide. Using DAR-5 from two-time points, Adler et al. [24] investigated to measure the problematic anger experienced in individuals and occupations by 90,266 U.S. military who were in the Army or Marines, active duty, and previously deployed with high levels of combat. Additionally, DAR-5 was used by surveying 736 residents of rural communities five years after the 2009 Black Saturday bushfires in Victoria, Australia, to measure the level of anger of those who have experienced wildfires due to natural disasters and how anger experience had affected their life [25]. DAR-5 was also used by surveying 5,114 UK adults experiencing lockdown due to the COVID-19 pandemic resulting in numerous confirmed cases and deaths to explore the correlations between anger and COVID-19 vaccine hesitancy [26]. Some studies have reported that DAR-5 has been employed in many populations exposed to trauma, such as nurses exposed to workplace violence [27] and individuals in need of mental health services [28].

Although the DAR-5 has proved its utility as a valid instrument for assessing problematic anger reactions, the scale has only been validated in three countries. The validation studies of the DAR-5 were conducted using samples of American college students with a subgroup of trauma-exposed individuals [3], community-dwelling French-speaking adults with prior trauma exposure who were mostly Swiss or French citizens [29], and Arabic-speaking adults and adolescents who had been experiencing military conflict since 2011, including those still living in Syria and those who fled the country as refugees [30]. However, the DAR-5 has been increasingly used in a variety of trauma-exposed populations, which includes trauma-exposed community-dwelling adults [31], the US military [24], mental health nurses who experienced workplace violence [27], and residents of areas affected by bushfires [25]. Despite its utility as a concise measure of anger that can be used on trauma-exposed populations, it is apparent that there is a lack of cross-cultural validation studies of the DAR-5.

For examining problematic anger that is associated with functional impairment by utilizing the DAR-5, a cut-off score of 12 on the DAR-5 was established for the normal college student sample [3]. This cut-off was then confirmed in the study with a sample of community-dwelling French-speaking adults [29]. Studies that utilized the DAR-5 have reported that the individuals with scores above 12 on the DAR-5 displayed higher scores with respect to PTSD symptoms, depression, anxiety, general psychological distress, and alcohol use [4, 29–31].

Given the numerous traumatic national events, which include the Daegu subway fire, Sewol ferry disaster, Pohang earthquake, and the bushfire, that have recently occurred in South Korea and the various subsequent studies on the negative impacts of these traumatic events, a concise and readily accessible scale of anger that has been validated for use in the South Korean population is necessary. However, the South Korean versions of the STAXI-1 (STAXI-1-K) for adults [32] and adolescents [33] are currently the only adapted instruments for assessing anger that are in practical use in the South Korean population. Additionally, the STAXI-2 has not yet been validated in the country, mainly due to the financial burden to obtain a copyright for its use. Therefore, it is valuable to validate the South Korean version of the DAR-5 (DAR-5-K), as it would significantly facilitate the assessment of anger in trauma-related clinical and research settings in the South Korean context. The South Korean adaptation of the DAR-5 would further strengthen the evidence for cultural generalization of the DAR-5 because it would examine the psychometric properties of the scale in the South Korean population.

## Purpose of the present study

The present study primarily aimed to demonstrate a factor structure of the DAR-5 in the South Korean context by using an exploratory factor analysis (EFA) and confirmatory factor analysis (CFA). Additionally, it aimed to expand on the psychometric properties that were previously established through the English, French, and Arabic versions of the DAR-5 in a community-dwelling sample of South Korean adults who experienced traumatic events. Thus, the internal reliability and concurrent validity of the DAR-5-K were evaluated. The concurrent validity of the scale was examined in relation to other variables, including PTSD symptoms, depression, anxiety, and self-esteem. Lastly, the present study aimed to apply a cut-off score of 12 on the DAR-5—scores above which have been reported to demarcate problematic anger in previous studies—to the South Korean adult sample.

## Method

### Participants

In the present study, a nationwide anonymous online survey was conducted in 2018 according to the South Korean population census standard. The survey accounted for socio-demographic factors such as age, gender, and residential area. A total of 1,657 individuals participated in the survey, and 1,137 (68.6% of the total) of them completed the survey. Of these 1,137, those who had not experienced a traumatic event (based on DSM-5 diagnostic criteria) (N = 323) were excluded. Therefore, the final sample consisted of 814 South Korean adults who had experienced traumatic events. All the participants were native Korean speakers.

### Descriptive population statistics

The mean age of the final sample (N = 814), which consisted of individuals ranging from 20 to 55 years of age, was 39.41 years (*SD* = 9.56). There were 174 participants who were in their 20s (21.4%), 221 in their 30s (27.1%), 270 in their 40s (33.2%), and 149 in their 50s (18.3%). In all, 421 (51.7%) participants were male and 393 (48.3%) were female. More than half of the respondents (N = 419, 51.5%) resided in Seoul and its surrounding metropolitan area. In terms of marital status, the participants were categorized as either married (N = 466, 57.2%), single (N = 319, 39.2%), and divorced/bereaved and others (N = 29, 3.5%). Most participants were employed (N = 546, 67.1%). Housewives (N = 67, 8.2%), professional workers (N = 55, 6.8%), self-employed individuals (N = 50, 6.1%), college students (N = 50, 6.1%), and unemployed/other individuals (N = 46, 5.7%) were also present in the sample. The participants marked one of the following categories as their educational level: middle school (N = 2, 0.2%), high school (N = 95, 11.7%), college (N = 614, 75.4%), and graduate (N = 102, 12.6%). The participants were categorized into two groups based on their monthly income. Among them, 397 (48.8%) earned less than the average monthly income of South Korean laborers (approximately USD 2,627), 394 (48.4%) earned equal to or more than it, and 23 (2.8%) chose ‘not applicable’.

### Trauma exposure

Table 1 presents the traumatic events listed on the Life Events Checklist-5 for DSM-5 (LEC-5) [34] and the number and percentage of people who reported these traumatic events as the most painful event that they experienced. The most commonly reported traumatic event was ‘any other very stressful event or experience.’ Other commonly reported traumatic events included transportation accidents, physical assault, life-threatening illnesses or injuries, and natural disasters.

**Table 1:** LEC-5 trauma types and scores of PC-PTSD-5 and DAR-5-K for each type of trauma (N = 814)

LEC-5 trauma types	N	%	PC-PTSD-5	DAR-5-K
			*M(SD)*	*M(SD)*
Any other very stressful event or experience	587	72.11	1.44 (1.61)	10.45 (4.94)
Transportation accident (for example, car accident, boat accident, train wreck, plane crash)	66	8.11	1.53 (1.55)	11.62 (5.60)
Life-threatening illness or injury	27	3.32	1.70 (1.77)	11.26 (6.12)
Physical assault (for example, being attacked, hit, slapped, kicked, beaten up)	28	3.44	1.68 (1.61)	11.25 (4.63)
Natural disaster (for example, flood, hurricane, tornado, earthquake)	19	2.33	1.16 (1.21)	10.68 (4.78)
Fire or explosion	15	1.84	1.53 (1.64)	12.47 (5.05)
Sudden violent death (for example, homicide, suicide)	13	1.6	2.23 (1.48)	9.46 (4.16)
Sudden accidental death	16	1.97	1.56 (1.31)	11.81 (5.13)
Serious accident at work, home, or during recreational activity	12	1.47	1.33 (1.72)	8.58 (3.12)
Sexual assault (rape, attempted rape, made to perform any type of sexual act through force or threat of harm)	11	1.35	1.27 (1.49)	10.64 (4.65)
Other unwanted or uncomfortable sexual experience	9	1.11	2.67 (1.22)	12.89 (4.68)
Assault with a weapon (for example, being shot, stabbed, threatened with a knife, gun, bomb)	3	0.37	2.67 (2.52)	10.67 (1.53)
Severe human suffering	2	0.25	2.00 (1.41)	16.00 (1.41)
Serious injury, harm, or death that you caused	2	0.25	2.50 (2.12)	13.00 (7.07)
Exposure to toxic substances (for example, dangerous chemicals, radiation)	2	0.25	4.50 (0.71)	15.00 (2.83)
Combat or exposure to a warzone (in the military or as a civilian)	1	0.12	0.00 (-)	5.00 (-)
Captivity (for example, being kidnapped, abducted, held hostage, prisoner of war)	1	0.12	0.00 (-)	5.00 (-)

LEC-5: Life Events Checklist-5. PC-PTSD-5: Primary Care PTSD Screen for DSM-5. DAR-5-K: South Korean version of the DAR-5

### Procedure

The survey was conducted in 2018 for about one month via an Internet-based survey company in South Korea. The survey company utilized firewall (WAF) and DigiCert security service—a digital certificate association that provides services certified by ISO 9001—to ensure security and the encryption of surveys. All responses from the survey were collected with encrypted secure sockets layer (SSL) connections to ensure the secure transmission of sensitive data. Users created their own passwords and entered their own username and password to log in, which ensured user authentication. The survey included questions that queried participants’ socio-demographic characteristics as well as multiple instruments for examining the sociological and psychological constructs of the respondents. The survey took approximately 20 min to complete. The participants were given some amount of online credit points as compensation. The present study was approved by the Institutional Review Board (IRB) of the university that the researchers are affiliated with.

### Instruments

Five instruments were used in order to cross-culturally validate the DAR-5. They are listed below.

#### LEC-5 trauma types

The LEC-5 [34] assesses exposure to 17 traumatic events. Participants report their exposure to each of the traumatic events by choosing one of the six possible answers: “happened to me,” “witnessed it,” “learned about it,” “part of my job,” “not sure,” or “does not apply to me.” The present study only included “happened to me” as a response. The participants were then asked to choose the most painful event that they experienced.

#### South Korean adaptation of the DAR-5

The DAR-5 [3] is composed of five items that measure anger frequency, intensity, duration, aggression, and its interference with social relations. The items are rated based on the respondent’s experience of anger over the past four weeks. Each item is scored on a 5-point Likert scale (1 = *none of the time*, 2 = *a little of the time*, 3 = *some of the time*, 4 = *most of the time*, 5 = *all of the time*), and the total scale score ranges from 5 to 25. Higher scores indicate a worse symptomatology of anger. Examples of items include, “I found myself getting angry at people or situations” and “When I got angry at someone, I wanted to hit them.” The DAR-5 was first translated from English to Korean by some bilingual researchers, including a psychology professor affiliated with a University in the United States. The instrument was then back-translated into English by a South Korean professor of Counseling Psychology Education and three researchers—one of whom was in a Ph.D. program and two of whom were in a master’s program in the field of Psychology. The research team members compared the back-translated version with the original English version of the scale until all the researchers reached a final agreement. A readability test of the DAR-5-K was conducted with high-school students and teachers in 2015. Their feedback was incorporated until there were no complaints regarding difficulties in reading or comprehending the items, and this finalized version of the DAR-5-K was used in the present study.

The original English version of the DAR-5 has been found to have good internal consistency with a Cronbach’s α of 0.89 to 0.90 [3]. Additionally, the French version of the scale had a Cronbach’s α of 0.80 [29] and the Arabic version had a Cronbach’s α of 0.72 to 0.83 [30]. The DAR-5 presented a single factor of anger experience in these previous validation studies and was established with convergent, discriminant, and concurrent validity.

#### Posttraumatic stress disorder symptoms

The Primary Care PTSD Screen for DSM-5 (PC-PTSD-5) [35] is a 5-item self-report measure that assesses the presence of PTSD symptoms based on the DSM-5 criteria. The PC-PTSD-5 has a dichotomous response format of either 1 (Yes) or 0 (No), and the total score ranges from 0 to 5. Examples of the items include, “Have you had nightmares about the event(s) or thought about the event(s) when you did not want to?” and “Have you been constantly on guard, watchful, or easily startled?” Higher scores indicate a higher risk of PTSD symptomatology. A cut-off score of 3 was used for the South Korean version of the PC-PTSD-5 in the present study to distinguish the group with higher PTSD symptoms from that with no/lower PTSD symptoms [36]. The higher PTSD symptoms group consisted of individuals with PC-PTSD-5 scores equal to or more than 3. The South Korean version of the PC-PTSD-5 showed acceptable internal consistency with a Cronbach’s α of 0.73 [36]. In the present study, the scale had a Cronbach’s α of 0.752.

#### Higher/no or lower PTSD symptoms group

As per the cut-off score of 3 that was determined in the validation study of the PC-PTSD-5 [35] and confirmed by Jung et al., [36] in the South Korean validation study of the scale, the present study divided the sample (N = 814) into two subgroups: the higher PTSD symptoms group (N = 216, 26.5%) and the no/lower PTSD symptoms group (N = 598, 73.5%).

#### Depression and anxiety symptoms

The Brief Symptom Inventory-18 (BSI-18) [37] is an 18-item self-report inventory that measures psychological distress in three domains: depression, anxiety, and somatization. Each item is scored on a 5-point Likert scale that ranges from 0 (not at all) to 4 (very much). The total scale score ranges from 0 to 72, with higher scores indicating higher levels of distress. Examples of items include, “Feeling hopeless about the future” (depression), “Nervousness or shakiness inside” (anxiety), and “Feeling weak in parts of your body” (somatization). Only two subscales that each measure depression and anxiety were utilized in the current study. The South Korean version of the BSI-18 [38] was shown to have good internal consistency with a Cronbach’s α of 0.80 for depression and 0.81 for anxiety. In the current study, the Cronbach’s α was 0.905 for depression and 0.920 for anxiety.

#### Self-esteem

The Rosenberg Self-esteem Scale [39] is a 10-item self-report questionnaire that measures global self-esteem. Each item is scored on a 4-point Likert scale that ranges from 1 (strongly disagree) to 4 (strongly agree). The total scale score ranges from 10 to 40, with higher scores indicating higher self-esteem. Sample items include, “On the whole, I am satisfied with myself” and “I feel that I have a number of good qualities.” The South Korean version of the Rosenberg Self-esteem Scale was demonstrated to have good internal consistency with a Cronbach’s α of 0.79 to 0.83 [40], and the scale in the present study had a Cronbach’s α of 0.831.

### Data analysis

814 participants were randomly divided into two data sets. One for EFA and another for CFA. EFA was first performed with a randomly selected half of the overall sample (N = 405), using maximum likelihood factoring with direct oblique rotation to examine the factor structure of the DAR-5-K. IBM SPSS Statistics 21.0. was used. First, the Kaiser-Meyer-Olkin (KMO) test and Bartlett’s test of sphericity were used to evaluate the sampling adequacy. Scree plot and factor interpretability were used to determine the number of factors, and factor loadings equal to or more than 0.40 were considered acceptable. Then, CFA with the other half of the sample (N = 409) was conducted with Mplus 8.0 to test the one-factor structure of the DAR-5-K items. Error terms were set to be uncorrelated. The CFA result was further validated with two additional samples, including the higher PTSD symptoms group, and the no/lower PTSD symptoms group. Since chi-square (${x}^{2}$) has been found to be extremely sensitive to sample size, fit statistics were derived to assess how well the one-factor structure of the DAR-5-K fits the data. The present study reported incremental fit statistics such as the comparative fit index (CFI), Tucker–Lewis index (TLI), standardized root mean square residual (SRMR), and the root mean square error of approximation (RMSEA). For a model to be considered good-fitting, it is suggested that the CFI and TLI must be > 0.95 and the SRMR must be < 0.08 [41]. An RMSEA of < 0.08 is considered a good fit [42].

In the present study, reliability analysis was conducted using IBM SPSS Statistics 21.0. Cronbach’s α was used to evaluate the internal consistency of each instrument. Composite Reliability (CR)—another measure of internal reliability was calculated. Finally, McDonald’s coefficient omega (ω)—an additional indicator of internal reliability that performs better than Cronbach’s alpha and is thus preferred when tau-equivalence is violated [43]—was also calculated. A Cronbach’s α, CR, and McDonald’s omega (ω) of 0.70 and above are considered to indicate good reliability. Further, t-tests were performed to identify differences between the groups divided by the PC-PTSD-5 scores (higher and no/lower PTSD symptoms groups) and the DAR-5-K scores (high and low DAR-5-K subsamples). The effect sizes were calculated with Cohen’s *d*. Cohen suggested that a Cohen’s *d* of 0.20, 0.50, and 0.80 should be considered small, medium, and large, respectively [44]. A t-test between the higher and no/lower PTSD symptoms groups was conducted only when factorial invariance was supported. The concurrent validity was assessed by examining the differences between the higher PTSD symptoms group and no/lower PTSD symptoms group in terms of the total DAR-5-K, depression, anxiety, and self-esteem scores, and correlations between the DAR-5-K and these other variables. Differences between the high and low (based on the cut-off) DAR-5-K subsamples in terms of the total PTSD symptoms, depression, anxiety, and self-esteem scores were examined. The cut-off DAR-5 score of 12 was determined in the original English validation study [1] and subsequently corroborated in the French validation study [29]. In the present study, an analysis of the Receiver Operating Characteristics curve (ROC curve) as a statistical tool to confirm this cut-off score of the DAR-5 could not be performed, as there was no other South Korean-validated measure of anger available with an identified cut-off score. Thus, this study applied the previously identified DAR-5 cut-off score to the current sample, as was also done in the Arabic validation study of the DAR-5 [30].

## Results

The means and standard deviations for the DAR-5-K, PC-PTSD-5 (PTSD symptoms), BSI-18 (depression, anxiety), and RSE (self-esteem) are presented in Table 2. The total score of the DAR-5-K approximated a normal distribution with a skewness value of 0.629 (*SE* = 0.086) and a kurtosis value of -0.479 (*SE* = 0.171). Other scales also followed a normal distribution; PC-PTSD-5 had a skewness value of 0.779 and a kurtosis value of -0.602; depression had 0.519 and − 0.507 skewness and kurtosis, respectively; anxiety had 0.729 and − 0.372 skewness and kurtosis, respectively; and lastly, self-esteem had a skewness value of -0.159 and a kurtosis value of -0.274.

**Table 2:** Mean scores for DAR-5-K, PC-PTSD-5 (PTSD symptoms), BSI-18 (Depression, Anxiety), and RSE (self-esteem) for overall sample (N = 814), higher PTSD symptoms group (N = 216), and no/lower PTSD symptoms group (N = 598)

Measure	Overall	Higher PTSD	No/lower PTSD	t-test	Cohen’s d
	*M(SD)*	*M(SD)*	*M(SD)*		
DAR-5-K Total^a^	10.68 (5.00)	13.48 (5.23)	9.66 (4.51)	9.52***	0.78
PTSD symptoms^b^	1.50 (1.60)	3.82 (0.80)	0.66 (0.79)	50.08***	3.95
Depression^c^	7.87 (5.97)	11.35 (6.13)	6.61 (5.39)	10.05***	0.82
Anxiety^c^	6.42 (5.76)	10.24 (6.06)	5.04 (4.98)	11.32***	0.94
Self-esteem^d^	28.48 (4.88)	26.28 (4.65)	29.28 (4.72)	-8.04***	0.64

^a^ DAR-5-K: South Korean version of the DAR-5. ^b^ PC-PTSD-5: Primary Care PTSD Screen for DSM-5. ^c^ BSI-18: Brief Symptom Inventory-18. ^d^ RSE: Rosenberg Self-esteem Scale

***p < .001

## Factor structure of the DAR-5-K

**Fig. 1:**
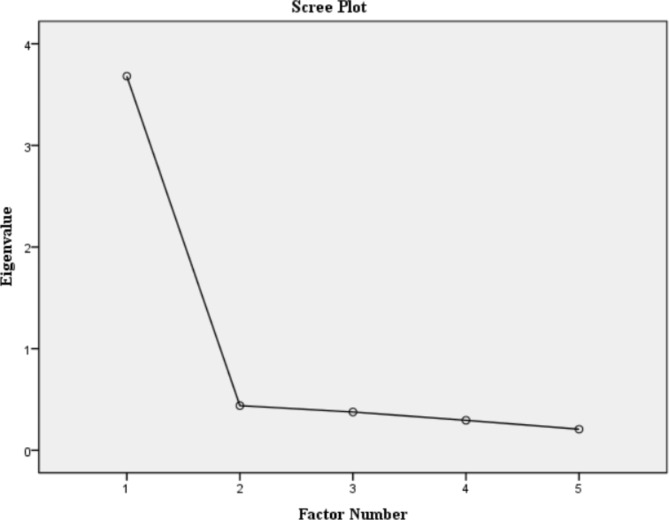
Results of the scree plot for the overall sample (N = 814)

Studies of the original English version [3], French version [29], and Arabic version [30] of the scale supported a one-factor structure for the DAR-5. Concerning EFA, KMO of 0.878 and Bartlett test results of p = .000 (${x}^{2}$=1321.288, df = 10) indicated that the data are suitable for factor analysis. Based on the scree plot (see Fig. 1) and the factor interpretability reported in the previous studies [3, 29], it was suggested to extract one factor for the DAR-5-K. The one-factor model explained 73.641% of the variance in the overall sample (N = 814), 71.199% in the higher PTSD symptoms group (N = 216), and 70.127% in the no/lower PTSD symptoms group (N = 598). The factor loadings from the EFA were as follows: item 1 (anger frequency) = 0.868, item 2 (anger intensity) = 0.864, item 3 (anger duration) = 0.830, item 4 (antagonism towards others) = 0.795, item 5 (social relations interference) = 0.733.

**Fig. 2:**
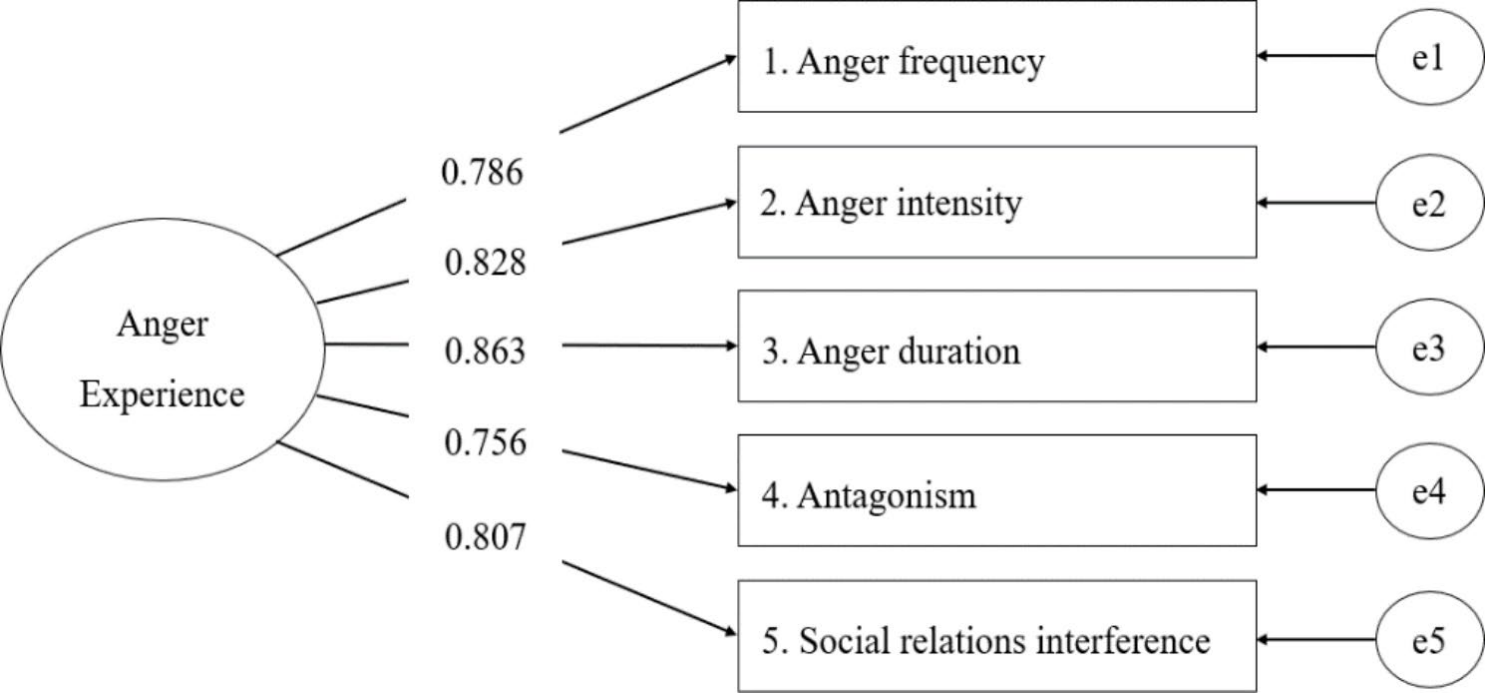
Results of the Confirmatory Factor Analysis of the DAR-5-K (N = 409)

The CFA for a one-factor model showed a satisfactory model fit, with a ${x}^{2}$= 5.208 (*df* = 5), *p* = .3910, CFI = 1.000, TLI = 1.000, SRMR = 0.009, and RMSEA = 0.010. For further validation of the CFA findings, the one-factor model was retested with two additional samples, including the higher PTSD symptoms group (N = 216) and no/lower PTSD symptoms group (N = 598). The higher PTSD symptoms group showed an acceptable model fit of ${x}^{2}= 11.137$(*df* = 5), *p* = .0487, CFI = 0.981, TLI = 0.963, SRMR = 0.024, and RMSEA = 0.105 (N = 216), and the no/lower PTSD symptoms group showed an acceptable model fit of${x}^{2}= 11.100$(*df* = 5), *p* = .0494, CFI = 0.993, TLI = 0.985, SRMR = 0.016, and RMSEA = 0.064. The standardized factor loadings for the five DAR-5-K items are presented in Fig. 2.

## Reliability

The DAR-5-K was shown to have a good internal consistency, as demonstrated by a Cronbach’s α of 0.893 to 0.907, CR of 0.893 to 0.907, and McDonald’s coefficient omega (ω) of 0.894 to 0.907. Excellent item-total correlations were observed with the DAR-5-K, with correlations ranging from 0.825 to 0.876 in the overall sample, 0.815 to 0.866 in the higher PTSD symptoms group, and 0.802 to 0.882 in the no/lower PTSD symptoms group. Inter-item correlations of the scale seemed adequate, ranging from 0.613 to 0.756 in the overall sample, 0.503 to 0.684 in the higher PTSD symptoms group, and 0.553 to 0.758 in the no/lower PTSD symptoms group. The PC-PTSD-5 (PTSD symptoms), BSI-18 (depression, anxiety), and RSE (self-esteem) also showed good internal consistency in the overall sample with Cronbach’s alphas of 0.752, 0.905, 0.920, and 0.831, respectively.

## Concurrent validity

Results of a Multigroup CFA across PTSD symptoms severity indicated that a metric invariance of the DAR-5-K was achieved. Fit indices of the configural model of the DAR-5-K were ${x}^{2}$(df) = 48.066(10), CFI = 0.983, TLI = 0.967, SRMR = 0.020, RMSEA = 0.097; those of the metric model were ${x}^{2}$(df) = 58.092(14), CFI = 0.981, TLI = 0.973, SRMR = 0.038, RMSEA = 0.088. Because the ${x}^{2}$ statistic is strongly affected by sample size, the CFI difference (ΔCFI) was used in the present study. The CFI difference between the configural model and metric model of the DAR-5-K was − 0.002, meeting the cut-off criterion (ΔCFI < 0.01) suggested by [45].

As presented in Table 2, individuals in the higher PTSD symptoms group reported significantly higher scores in terms of anger reactions (*t* = 9.517, *p* < .001), PTSD symptoms (*t* = 50.082, *p* < .001), depression (*t* = 10.054, *p* < .001), and anxiety (*t* = 11.317, *p* < .001), and lower scores with respect to self-esteem (*t* = -8.044, *p* < .001) compared to individuals in the no/lower PTSD symptoms group. The higher and no/lower PTSD symptoms groups differed with a medium to large effect size (Cohen’s *d* from 0.64 to 3.95). A large effect size was found for PTSD symptoms (*d* = 3.95), depression (*d* = 0.82), and anxiety (*d* = 0.94), and a medium effect size was found for anger reactions (*d* = 0.78) and self-esteem (*d* = 0.64).

**Table 3:** Correlations between the DAR-5-K and the following measures: BSI-18 (depression, anxiety) and RSE (self-esteem)

	Overall (N = 814)	Higher PTSD symptoms (N = 216)	No/lower PTSD symptoms (N = 598)
Depression^a^	0.733^***^	0.725^***^	0.684^***^
Anxiety^a^	0.761^***^	0.741^***^	0.718^***^
Self-esteem^b^	-0.390^***^	-0.331^***^	-0.330^***^

DAR-5-K: South Korean version of the DAR-5. ^a^ BSI-18: Brief Symptom Inventory-18. ^b^ RSE: Rosenberg Self-esteem Scale

***P < .001

Correlations between the DAR-5-K total scores and the depression, anxiety, and self-esteem scores are presented in Table 3. All correlations between the DAR-5-K and other variables were significant, with depression ranging from 0.684 to 0.733 (p < .001), anxiety ranging from 0.718 to 0.761 (p < .001), and self-esteem ranging from − 0.330 to -0.390 (p < .001).

## DAR-5-K cut-off score

Table 4 shows the differences in mean scores of PTSD symptoms, depression, anxiety, and self-esteem between the high and low DAR-5-K subsamples. A cut-off score of 12 was used in the present study to divide the high and low DAR-5 subsamples, as scores above 12 indicated the presence of problematic anger in previous validation studies [3, 29]. Individuals in the high DAR-5-K subsample showed higher scores for PTSD symptoms (*t* = 9.913, *p* < .001), depression (*t* = 23.484, *p* < .001), and anxiety (*t* = 23.432, *p* < .001), and lower scores for self-esteem (*t* = -9.497, *p* < .001). The high and low DAR-5-K subsamples differed with a medium to large effect size (Cohen’s *d* from 0.68 to 1.73). A large effect size was found for depression (*d* = 1.70) and anxiety (*d* = 1.73), and a medium effect size was found for PTSD symptoms (*d* = 0.72) and self-esteem (*d* = 0.68).

**Table 4:** Mean scores of PC-PTSD-5 (PTSD symptoms), BSI-18 (depression, anxiety), and RSE (self-esteem) for the overall sample (N = 814), high DAR-5-K subsample (N = 330), and low DAR-5-K subsample (N = 484) (cut-off point = 12)

Measure	Overall	High DAR-5-K	Low DAR-5-K	t-test	*Cohen’s d*
	*M(SD)*	*M(SD)*	*M(SD)*		
PTSD symptoms^a^	1.50(1.60)	2.16(1.72)	1.05(1.34)	9.913***	0.72
Depression^b^	7.87(5.97)	12.56(5.03)	4.67(4.19)	23.484***	1.70
Anxiety^b^	6.42(5.76)	11.03(5.23)	3.28(3.59)	23.432***	1.73
Self-esteem^c^	28.48(4.88)	26.62(4.62)	29.76(4.65)	-9.497***	0.68

DAR-5-K: South Korean version of the DAR-5. ^a^ PC-PTSD-5: Primary Care PTSD Screen for DSM-5. ^b^ BSI-18: Brief Symptom Inventory-18. ^c^ RSE: Rosenberg Self-esteem Scale

***p < .001

## Discussion

Anger is one of the commonly reported symptoms among populations that have experienced traumatic events. Despite the numerous traumatic events that have occurred in recent years in South Korea and the presence of research on the anger that emerges due to such traumatic events, a concise and accessible measure of anger has yet to be validated in the South Korean setting. Therefore, the present study conducted the South Korean validation of the DAR-5, and demonstrated the DAR-5-K to be a concise, valid, and reliable measure for screening anger among South Korean adults who have had traumatic experiences. Comprising only five items, the DAR-5-K demonstrated good internal reliability (α = 0.893 to α = 0.907, CR = 0.893 to CR = 0.907, ω = 0.894 to ω = 0.907), good item-total correlations (*r* = .825 to *r* = .876), and moderate inter-item correlations (*r* = .613 to r = .756) in the overall sample. In this study, a one-factor structure of the scale was confirmed, as reported in the English, French, and Arabic validation studies of the DAR-5.

In this study, the CFA results demonstrated the replicability of the factor structure of the original DAR-5 with five items. A CFA with the DAR-5-K showed an excellent model fit with an overall sample in measuring the anger construct (${x}^{2}$= 5.208 (*df* = 5), CFI = 1.000, TLI = 1.000, SRMR = 0.009, RMSEA = 0.010). However, the RMSEA score (0.105) of the higher PTSD symptoms group revealed the need for further validation and item refinement. This indicated that the nature of anger demonstrated in the higher PTSD symptoms group could have been distorted by their experience of PTSD. In general, anger is a normal and natural response to a certain situation. However, PTSD can add another layer to one’s emotion as the function of one’s anger may be widely different depending on the context of one’s PTSD. This poor model fit with the higher PTSD symptoms group can also be cultural. Identifying possible reasons that contribute to the poor model fit is beyond the scope of the present study. However, these results warrant future study.

Further explanations regarding what could have contributed to a poorer RMSEA value in the higher PTSD symptoms group can be explored. One possible explanation is that it could have resulted from the small sample size. As [46] stated, “when sample size is low, RMSEA may suggest rejecting a model that otherwise would be accepted.” Although the CFI, TLI, and SRMR values were invariably great in the overall sample as well as in both subgroups, the RMSEA value was not particularly great in the higher PTSD symptoms group, which had the smallest sample size (N = 216). The overall sample (N = 814, RMSEA = 0.010, CFI = 1.000) and no/lower PTSD symptoms group (N = 598, RMSEA = 0.064, CFI = 0.993) had RMSEA values well below 0.08 with excellent CFI values, whereas the higher PTSD symptoms group (N = 216, RMSEA = 0.105, CFI = 0.981), which had the smallest sample size, displayed a rather poor RMSEA but an excellent CFI value. Another possible explanation for the poor RMSEA in comparison to the CFI, TLI, and SRMR can be the small degree of freedom (df; df = 5) in the present study. This causation is found in the following formula: population value of the RMSEA = $\sqrt{\frac{{\text{F}}_{0}}{\text{d}\text{f}}}$ [47, 48] (“${\text{F}}_{0}$ represents a suitably weighted sum of squared deviations between the population covariance matrix and the covariance matrix implied by the best fit of the hypothesized model to the population covariance matrix” [49]). Based on these findings, we took the values of CFI > 0.95, TLI > 0.95, and SRMR < 0.08 in this study as indicators of good model fit.

Concurrent validity was established based on the result that individuals in the higher PTSD symptoms group displayed significantly higher levels of anger reactions, depression, and anxiety than those in the no/lower PTSD symptoms group. This result replicated the findings obtained from the original validation study of the DAR-5 where significant differences in the levels of anger reactions on the DAR-5 between groups of individuals with high and low PTSD symptoms were found [3]. This result supported the French validation study in which there were significant differences in the DAR-5, depression, and anxiety scores between a high trauma group and low trauma group, which were constructed based on the number of traumatic event types on the LEC-5 that each respondent was exposed to [29].

The concurrent validity of the DAR-5-K was also supported by the significant correlations of anger reactions with depression and anxiety in the overall sample, higher PTSD symptoms group, and no/lower PTSD symptoms group. Again, these results replicated the findings obtained during previous validation studies of the DAR-5 [3, 29]. Further, based on the previous findings that individuals in the PTSD group had lower self-esteem than those in the no-PTSD group [50] and that anger showed a significant correlation with self-esteem [51], the self-esteem variable was also used in this study when examining the concurrent validity of the DAR-5-K.

Forbes et al. [3] determined a DAR-5 cut-off point of 12, which represented the 75th percentile in a sample of college students with and without trauma exposure. This was based on the idea of establishing a cut-off score of 21 for Trait Anger on the STAXI-2; a cut-off point of 21 has been found to place normal adult respondents at the 75th percentile [21]. In the original English validation study, individuals with scores above 12 on the DAR-5 were considered to experience problematic anger that is associated with functional impairment. In all, 125 individuals reported DAR-5 scores > 12 and were categorized into the high DAR-5 group, whereas 337 showed scores ≤ 12 and were included in the low DAR-5 group. In the French validation study of the DAR-5, 166 (20.2%) individuals reported DAR-5 scores > 12 and 656 (79.8%) individuals reported scores ≤ 12 [29]. This denoted that the cut-off score of 12 was located at around the 75th percentile of the study sample. This cut-off score was backed up by an analysis of the ROC curve that suggested that the cut-off point of 12 reasonably predicted a “clinical posttraumatic anger” [29]. In the Arabic validation study of the DAR-5, 566 (35.8%) adults reported DAR-5 scores ≥ 12 and 473 (60.3%) adolescents reported scores ≥ 12 [30].

The current validation study thus applied the cut-off point determined in these previous validation studies [3, 29] to the DAR-5-K with the sample of the South Korean adults exposed to traumatic events. In all, 330 (40.5%) adults were included in the high DAR-5-K group and 484 (59.5%) were categorized as the low DAR-5-K group. Individuals in the high DAR-5-K group with a clinical level of anger reported higher levels of PTSD symptoms, depression, and anxiety, and lower self-esteem than the other group, and medium to large effect sizes were found with the clinical cut-off point of 12 on the DAR-5-K. Consistent results have been previously reported in validation studies of the DAR-5, which have shown significant differences in PTSD symptoms, depression, and anxiety between the high and low DAR-5 groups when divided based on the aforementioned cut-off point [3, 29, 30].

The aforementioned findings related to reliability, factor structure, concurrent validity, and the results with a cut-off point for the DAR-5-K demonstrate that the scale is a reliable and valid tool to measure problematic anger following traumatic events. This suggests that the results of psychometric properties of the DAR-5 reported in previous validation studies [3, 29, 30] can be extrapolated to the South Korean adult population.

However, these conclusions should be taken into consideration with cognizance of the limitations of the present study. The present study categorized the overall sample into two groups, namely the higher and no/lower PTSD symptoms groups, which were divided based on a cut-off point of 3 on the PC-PTSD-5—an instrument for posttraumatic stress symptoms. However, a clinical sample diagnosed with psychopathologies was absent in the current study. The use of a clinical sample and implementation of diagnostic tools for psychiatric illnesses including PTSD, depression, and anxiety in future studies would strengthen the results of the current study. Further, in addition to the self-report measures used in the present study, the validity of the DAR-5-K could be improved through future research where observational reports of behavioral problems associated with anger are documented and a structured interview for the psychiatric disorders related to anger is administered.

Moreover, unlike the original English and French validation studies of the DAR-5, the present study does not use the STAXI-2 as a supplementary tool to confirm the cut-off score of 12. The use of the STAXI-2 with an established cut-off score was not possible as a validation study of the STAXI-2 has not yet been performed in the South Korean context. The current study did not make use of the South Korean version of the STAXI-1 (STAXI-1-K) either. This was because, to the best of our knowledge, a cut-off point for the STAXI-1-K has not been established. Further, given the number of items in the STAXI-1 (44 items) and STAXI-2 (57 items), an addition of a lengthy questionnaire would have significantly increased the cognitive burden on participants. This would consequently increase the survey dropout rate, as suggested by [52]. Therefore, based on previous validation studies of the DAR-5 [3, 29], we applied a cut-off point of 12 to the current study sample. However, when the South Korean validation of the STAXI-2 or that of an analogous anger scale with a cut-off score is conducted at some point in the future, a follow-up study should be conducted to decide whether it is appropriate to use a cut-off score of 12 with the South Korean population.

In conclusion, the present study indicates that the DAR-5-K has robust psychometric properties and is a shortened brief and effective measure to evaluate the presence of problematic anger in the South Korean adult population who has been exposed to traumatic events. The generalizability of the results obtained by using the DAR-5 in the previous validation studies [3, 29, 30] is supported in the sample of South Korean adults. Furthermore, the brevity of the scale renders it feasible to be included in the batteries of self-report measures.

## Data Availability

The datasets used and/or analyzed during the current study are available from the corresponding author upon reasonable request.
